# Effects of *Gossypium* spp., *Balanites aegyptiaca*, and *Sesamum indicum* seeds oils on quality of chilled and frozen-thawed ram semen

**DOI:** 10.14202/vetworld.2021.1412-1419

**Published:** 2021-06-03

**Authors:** Adamou Akourki, Arantxa Echegaray, Orlando Perdomo, Nicolas Angel Escartin, Marta Guillén

**Affiliations:** 1 Enseignant Chercheur, Université Dan Dicko Dankoulodo de Maradi, BP: 465 Maradi, Niger; 2 Departamento de Reproducción animal del HUMECO, C/Mecanica 11. 22006 Huesca. Spain

**Keywords:** *Balanites aegyptiaca*, *Gossypium* spp, ram, semen freezing, *Sesamum indicum*

## Abstract

**Background and Aim:**

Essential oils found frequently in plants are well known for their activities against bacteria, viruses, and fungi, and antioxidant properties. This study aimed to analyze egg yolk replacement by seed oils of *Gossypium* spp. (cotton), *Balanites aegyptiaca* (desert date), and *Sesamum indicum* (sesame) in semen extender, on ram sperm quality chilled at 4°C and frozen-thawed.

**Materials and Methods:**

Ejaculates were collected from adult rams and refrigerated at 4°C in a Tris-based extender containing 1.25%, 2.5%, 5%, and 10% of *Gossypium* spp., *B. aegyptiaca*, and *S. indicum* seed oils, to evaluate which were the two best extenders for comparison with BIOXcell, a commercial extender for deep freezing ram semen.

**Results:**

The data showed that sperm movements analyzed by the CASA system were faster in extenders supplemented with 2.5-5% of cottonseed oil and 1.25-10% of sesame oil, whereas in the extender containing *B. aegyptiaca* oil, all seminal parameters studied had the worst values. During the sperm-freezing process, 5% of cottonseed oil and 5% sesame seed oil were selected from the first study, with sesame oil reaching the best sperm quality. Thus, sperm motility and velocity were 44.14±13.99%, 24.44±12.6%, and 25.92±11.50%; and 20.26±9.56%, 8.76±6.38%, and 9.42±5.40%, respectively, for sesame oil, cottonseed oil, and BIOXcell.

**Conclusion:**

In summary, 2.5-10% of cottonseed oil and 1.25-10% of sesame seed oil can replace egg yolk in a Tris–egg yolk–based extender. Moreover, a Tris-based extender supplemented with 5% sesame seed oil could be an alternative for deep freezing ram semen, even though these results need to be confirmed with semen collected from rams with appropriate sexual rest.

## Introduction

Niger is a West African country whose animal husbandry contributes to nearly 12% of the national gross domestic product [1]. According to the last national livestock survey, Niger has 7,340,610 cows, 7,932,586 sheep, 11,241,280 goats, 1,565,421 camels, 232,470 horses, and 1,474,882 donkeys [2]. Among these species, small ruminants (goats and sheep) are the most numerous and contribute more to household sustainability. Breeders sell nearly 20% of the small ruminants annually to solve economic and social emergencies [3]. Two main breeds, the red goat of Maradi and Balami sheep, have been well researched in and outside Niger for their skin quality, meat, and high milk production. Most of these breeds’ flocks are concentrated in the country’s south-east.

Despite providing food security, small ruminants are still being raised by the extensive system and natural breeding, which does not ensure enough animal multiplication. If we want to continue providing enough income for Nigerien households and to satisfy the increasing market demand for the Balami sheep and Red goat of Maradi, it is vital to intensify production. Artificial insemination using chilled and frozen semen can accelerate animal production in breeder farms, simultaneously increasing genetic progress. Indeed, numerous studies have already demonstrated the efficiency of artificial insemination in small ruminants [4-11]. In a comparative study between extenders for freezing semen, a pregnancy rate of 54.55-63.64% was found in Rhamani ram [4], a lambing rate of 56.7-64.5% in Suffolk ewes [5], and a pregnancy rate of 46.2-71% of in Chios rams [6]. In goats, depending on the insemination protocol, fertility varied from 30.3% to 52.5% [7], 38-50% according to the frozen semen type used [8], 35-65% by the synchronization method used [9], 62-64.3% depending on the number of inseminations done [10], and 46-75% per time taken to inseminate a goat [11]. Most of the semen used is frozen in extenders containing animal substances such as lecithin protein [8,12], increasing the risk of the spread of animal diseases. It is, therefore, essential to find safer sperm-freezing alternatives. Recently, studies have focused on testing alternative extenders free of animal substances, such as soybean lecithin [4,5,6,13-17] or extenders supplemented with polyunsaturated fatty acids [18,19] or with vegetable oils, such as *argan oil*, *olive oil*, *Rosmarinus officinalis* oil, *Nigella sativa* oil, and Origanum vulgare oil [20-26] to preserve animal sperm. Other vegetables such as *Gossypium* spp., *Balanites aegyptiaca*, and *Sesamum indicum*, available widely in Africa, including Niger, also contain lecithin protein and unsaturated fatty acids [27-29], but have not yet been tested for sperm cryopreservation. Then, because of its essential fatty acids’ composition, it was hypothesized that *Gossypium* spp., *B. aegyptiaca*, and *S. indicum* seed oil incorporated into sperm extender could minimize spermatozoa damage associated with cold shock and thawing stress during the sperm freezing-thawing process.

The present study aimed to analyze egg yolk replacement by *Gossypium* spp., *B. aegyptiaca*, or *S. indicum* seed oils in semen extender on ram sperm quality chilled at 4°C and frozen-thawed.

## Materials and Methods

### Ethical approval

All experimental protocols and animal care were approved by the Spanish Committee of Ethics in Animal Experimentation as established by the Royal Decree 1386/2018 of November 19.

### Study period and location

This study took place from March 23 to May 20, 2019, at the Animal Reproduction Laboratory of HUMECO Company, Huesca (Spain).

### Experimental protocols

The study was performed in two experiments.

The first experiment’s purpose was to select new extenders containing oil obtained from *B. aegyptiaca* (desert date), *Gossypium* spp. (cotton), and *S. indicum* (sesame) seeds. Four concentrations (1.25%, 2.5%, 5%, and 10%) of desert date seed oil, cottonseed oil, and sesame seed oil were added to a Tris–fructose base extender (Table-1). These oil concentrations were chosen based on the De Valle team’s recommendations for freezing ram semen using palm and coconut oils [30].

**Table-1:** Composition of extenders used for sperm refrigeration (at 4°C) in Merino rams.

Control	OCS_1,25_	OCS_2,5_	OCS_5_	OCS_10_	ODDS_1,25_	ODDS_2,5_	ODDS_5_	ODDS_10_	OSS_1,25_	OSS_2,5_	OSS_5_	OSS_10_
3.03 g Tris	3.03 g Tris	3.03 g Tris	3.03 g Tris	3.03 g Tris	3.03 g Tris	3.03 g Tris	3.03 g Tris	3.03 g Tris	3.03 g Tris	3.03 g Tris	3.03 g Tris	3.03 g Tris
1.74 g citric acid	1.74 g citric acid	1.74 g citric acid	1.74 g citric acid	1.74 g citric acid	1.74 g citric acid	1.74 g citric acid	1.74 g citric acid	1.74 g citric acid	1.74 g citric acid	1.74 g citric acid	1.74 g citric acid	1.74 g citric acid
0.80 g fructose	0.80 g fructose	0.80 g fructose	0.80 g fructose	0.80 g fructose	0.80 g fructose	0.80 g fructose	0.80 g fructose	0.80 g fructose	0.80 g fructose	0.80 g fructose	0.80 g fructose	0.80 g fructose
20 mL Egg yolk	1.25 mL OCS	2.5 mL OCS	5 mL OCS	10 mL OCS	1.25 mL ODDS	2.5 mL ODDS	5 mL ODDS	10 mL ODDS	1.25 mL of OSS	2.5 mL of OSS	5 mL of OSS	10 mL of OSS
penicillin G 1000 UI/mL	penicillin G 1000 UI/mL	penicillin G 1000 UI/mL	penicillin G 1000 UI/mL	penicillin G 1000 UI/mL	penicillin G 1000 UI/mL	penicillin G 1000 UI/mL	penicillin G 1000 UI/mL	penicillin G 1000 UI/mL	penicillin G 1000 UI/mL	penicillin G 1000 UI/mL	penicillin G 1000 UI/mL	penicillin G 1000 UI/mL
streptomycin 10 mg/mL	streptomycin 1 mg/mL	streptomycin 1 mg/mL	streptomycin 1 mg/mL	streptomycin 10 mg/mL	streptomycin 1 mg/mL	streptomycin 1 mg/mL	streptomycin 1 mg/mL	streptomycin 1 mg/mL	Streptomycin 1 mg/mL	streptomycin 1 mg/mL	streptomycin 1 mg/mL	streptomycin 10mg/mL
80 mL distilled water	98.5 mL distilled water	97.5 mL distilled water	95 mL distilled water	90 mL distilled water	98.5 mL distilled water	97.5 mL distilled water	95 mL distilled water	90 mL distilled water	98.5 mL distilled water	97.5 mL distilled water	95 mL distilled water	90 mL distilled water

OCS_1,25_; OCS_2,5_; OCS_5_ and OCS_10_=Oil of cotton seeds at 1,25%; 2,5%; 5% and 10% in the extender. ODDS_1,25_; ODDS_2,5_; ODDS_5_ and ODDS_10_=Oil of desert date seeds at 1,25%; 2,5%; 5% y 10% in the extender. OSS_1,25_; OSS_2,5_; OSS_5_ and OSS_10_=Oil of sesame seeds at 1,25%; 2,5%; 5% y 10% in the extender. Tris=Tris-hydroxymethyl-aminomethane

For sperm collection, ejaculates were collected using an electro-ejaculator from 14 adult Merino rams aged 2.5±0.5 years, separated from sheep for only 3 days. Mixed semen was diluted in each extender before being kept in the refrigerator at 4°C for 3 days, where its quality was checked every 24 h for sperm motility. Each batch of the extender was replicated 6 times to evaluate sperm quality, and all animals used were owned by breeders from the Huesca region (Spain). It is important to note that in the experiment, Merino rams, which have a long bibliographic review in the manuscript, were used instead of the local Niger breed (Balami sheep), because the plant oils tested are found widely in Niger and because, in Spain where the study was conducted, Balami sheep are not breeding. The same experiment is now being developed on Balami sheep in Niger to confirm the results obtained.

The second experiment aimed to compare frozen semen’s quality with BIOXcell, a commercial extender, and two oil extenders with better results, selected in the first experiment (extenders with 5% of sesame oil and 5% cottonseed oil). A 6% glycerol solution was added to oil extenders and BIOXcell (Ref. 006584 IMV Technologies,L’ Aigle de Paris, France) following the supplier’s protocol for freezing semen. The semen was collected from the same rams as described in the first experiment and diluted progressively in each extender until a concentration of 400 million spermatozoa/mL was reached. After 4 h of cooling and stabilization at 4°C, straws were frozen in nitrogen vapor at 4 cm of nitrogen liquid level and stocked in nitrogen tanks. For sperm evaluation, straws were thawed at 37°C for 30 s and checked for sperm motility, viability, and acrosome integrity. Here also, six replicates of each diluent were used for the sperm freezing-thawing evaluation.

Desert date seed oil and sesame seed oil were purchased from local producers, Sahara Sahel Food^©^ (Zinder, Niger) and Union Faraâ (Tessaoua, Niger), respectively. Cottonseed oil was purchased from an https://www.amazon.com seller (Saaqin Inc., New York, USA) and the other chemicals used for extender preparation were bought from SIGMA-MERCK (Darmstadt, Germany). For the preparation methods, both desert date seed oil and sesame seed oil were extracted directly from selected seeds by the press extraction method without adding any solvent.

### Assessment of sperm motility

Sperm motility parameters were analyzed by a computer-assisted sperm motility analysis (SCOPUS, Version 1.0. HUMECO, Spain). Sperm samples were diluted to reach a sperm concentration of 25-50 million spermatozoa/mL and loaded onto a warmed (37°C) 20-mm Leja® 4-chamber slide. A minimum of three fields and 500 sperm tracks were evaluated at 100× for each sample chamber. The following variables were analyzed: Total motility (%); progressive motility (%); average path velocity (VAP, m/s); straight-line velocity (VSL, m/s); curvilinear velocity (m/s); straightness (%); and linearity (%).

### Assessments of sperm viability and acrosome integrity

Sperm viability and acrosome integrity were studied by flow cytometry using a Guava easyCyte 5HT (serial number: 673512S015, Millipore, IMV Technologies, L’Aigle, France**)** and an EASYKIT 5 viability and acrosome integrity (Ref. 025293, IMV Technologies, L’Aigle, France).

The staining protocol was as follows: First, the test tubes were removed from the package and placed on the working table; 200 mL of Easybuffer A (Ref. 022162, Lot 6111502, IMV Technologies, L’Aigle, France) and 2 mL of semen were added to each tube. After this, the plate was covered with a black lid and incubated at 37°C for 10 min and at room temperature for 15 min. Then, the samples were run through the flow cytometer with the “Easykit 5” taking 15 s to read each sample.

In addition, all spermatozoa were stained with yellow fluorochrome. This dye is used as a cell-permeable nucleic acid stain to label all the spermatozoa and remove debris from the analysis. Hence, spermatozoa with disrupted acrosomes are labeled with a green fluorochrome, and dead spermatozoa with damaged plasma membranes are labeled with a red fluorochrome.

### Statistical analysis

All the data collected were analyzed with the SPSS 20 Statistical software (IBM SPSS, Chicago, IL, USA) [31] for Windows, where a multivariate analysis of variance was performed to analyze the extenders’ effects on sperm motility, viability, and integrity. Duncan’s test for multiple comparisons was performed to detect the difference between arithmetic means of seminal parameters. Significant differences were considered when p<0.05.

## Results

### First experiment sperm conservation at 4°C

There was an extender effect on the parameters of sperm analyzed by the CASA system during sperm refrigeration at 4°C. Thus, the total motility mean was the highest in semen diluted with extender containing 1.25% of sesame oil (47.08±12.27%), even though the difference was not statistically significant (p=0.355) from the other extenders, except for those that contained desert date oil. For sperm progressive motility, the Tris–egg yolk extender control reached the highest values with 8.86±4.41% of motility average, but it is essential to state that this difference was not significant (p=0.08) when compared with most extenders, again, except for the extenders supplemented with 2.5-10% of desert date oil (Table-2).

**Table-2:** Mean values of sperm motility parameters during 3 days of semen preservation (at 4ºC) in Merino rams.

Extender	TM (%)	PM (%)	Fast movement (%)	Moderate movement (%)	Slow movement (%)	Static movement (%)
OCS_1,25_	37.4^bc^±9.9	5^abc^±3.2	16.8^abc^±6	13.2^ab^±4.6	52.9±10.8	17.2^ab^±4.3
OCS_2.5_	41.2^bc^±17.7	5.1^abc^±2.8	19.3^c^±8.9	13.7^ab^±6.5	49.1±9.6	17.9^ab^±7.3
OCS_5_	44.8^bc^±13.7	6.3^bc^±2.9	20.9^c^±8.23	15^b^±5.1	45.9±8.5	18.3^ab^±5.5
OCS_10_	39.0^bc^±16.9	5.1^abc^±1.8	18.2^bc^±9.8	13.1^ab^±6.4	48.5±1	20.3^ab^±7.5
ODDS_1.25_	24.5^ab^±19.6	5.7^abc^±5.8	11.4^abc^±10.7	11.4^ab^±9.1	44.8±10	32.4^ab^±20.7
ODDS_2.5_	18.7^a^±15.4	4.4^ab^±5.95	10.9^abc^±12.3	10.3^ab^±9.1	46.3±16.9	32.6^ab^±17.4
ODDS_5_	16.9^a^±16.9	3.2^ab^±3.4	7.3^ab^±6.8	8.5^ab^±8.4	53.2±17.4	31.1^ab^±19.1
ODDS_10_	11.4^a^±13	1.8^a^±2.8	5.6^a^±6.4	6^a^±6.7	51.9±18.1	36.5^b^±21.4
OSS_1.25_	47.1^c^±12.3	7.4^bc^±4.3	21.3^c^±9	15.6^b^±6.3	46±9.5	17.1^ab^±3.9
OSS_2.5_	41.8^bc^±17.4	6.1^bc^±3.9	20.1^c^±9.6	13.5^ab^±6	48.8±12.3	17.7^ab^±3.8
OSS_5_	42.4^bc^±13.8	6.8^bc^±3	22^c^±7.9	12.2^ab^±4.7	49±11.1	16.8^a^±5.1
OSS_10_	42.5^bc^±19.2	6.3^bc^±3.8	21^c^±11.9	12.7^ab^±4.9	48.9±12	17.3^ab^±5.3
TEY	39.5^bc^±6.8	8.9^c^±4.4	18.1^bc^±4.6	14.5^b^±2.1	49.4±3.2	18^ab^±4.2
P					0.370	

Different letters in the same column means statistical difference (p<0.05) between arithmetic means. OCS_1.25_; OCS_2.5_; OCS_5_; and OCS_10_=Oil of cotton seeds at 1.25%; 2.5%; 5% and 10% in the extender. ODDS_1.25_; ODDS_2.5_; ODDS_5_; and ODDS_10_=Oil of desert date seeds at 1.25%; 2.5%; 5% y 10% in the extender. OSS_1.25_; OSS_2.5_; OSS_5_ and OSS_10_=Oil of sesame seeds at 1.25%; 2.5%; 5% y 10% in the extender. TEY=Tris egg yolk base extender.TM=Total motility, PM=Progressive motility

In contrast, sperm movements were faster in extenders supplemented with 2.5-5% of cottonseed oil and 1.25-10% of sesame oil than extenders containing 5-10% of desert date oil. Furthermore, spermatozoa with moderate movements were more prevalent in extenders with 5% cottonseed oil, 1.25% of sesame oil, and in the control than in the extender containing 10% of desert date oil. Finally, static spermatozoa were more numerous in 10% of the desert date oil-based extender and less numerous in the extender containing 5% of sesame oil, with 36.5±21.4% and 16.8±5.05% of values, respectively (Table-2).

Average values of sperm velocity, especially VAP and VCL, were lower (p=0.015 and p=0.000, respectively) in semen diluted with desert date oil, mainly for concentrations of 2.5% and 10%. In contrast, extenders supplemented with sesame oil, notably those containing 1.25%, 5%, and 10% for VAP and 1.25%, 2.5%, and 5% for VCL reached the highest values (Table-3).

**Table-3:** Means values of sperm velocity parameters during semen conservation at 4°C in Merino rams.

Extender	VAP (mm/s)	VSL (mm/s)	VCL (mm/s)	LIN (%)
OCS_1,25_	25.9^abc^±10.5	10.4±5.1	70.3^cd^±12.7	12.7±5.8
OCS_2.5_	27.2^bc^±11.1	11.6±4.9	69^cd^±16.4	15.7±4.8
OCS_5_	27.5^bc^±11.4	12.1±5.1	71.2^cd^±14.9	14.45±5.43
OCS_10_	25.2^abc^±11.4	10.8±5.6	67^cd^±16	14.1±5.4
ODDS_1.25_	16.2^abc^±7.3	7.4±5.2	49.6^abc^±12.2	14.2±7.6
ODDS_2.5_	12.1^a^±5.2	5.8±4.4	42.5^a^±9.5	15.2±9.7
ODDS_5_	13.2^ab^±4.2	5.3±3.2	44.2^ab^±9.8	15.3±9.1
ODDS_10_	11.8^a^±5.1	5.8±3	35.7^a^±12.6	21.2±10.5
OSS_1.25_	28.9^c^±11.3	13.3±5	76.5^d^±15.8	13.9±4
OSS_2.5_	27.4^bc^±11.1	11.8±6.6	73.2^d^±18.1	134.8
OSS_5_	29.9^c^±9.8	13±5.5	75.1^d^±14.1	15.3±4.8
OSS_10_	28.2^c^±11.1	12.4±7.4	71^cd^±18	15.35
TEY	25^ab^±6.8	13.3±6.3	63.6^bcd^±5.2	19.5±7.7
p-value		0.069		0.123

Different letters in the same column means statistical difference (p<0.05) between arithmetic means. OCS_1.25_; OCS_2.5_; OCS_5_; and OCS_10_=Oil of cotton seeds at 1.25%; 2.5%; 5%; and 10% in the extender. ODDS_1.25_; ODDS_2.5_; ODDS_5_; and ODDS_10_=Oil of desert date seeds at 1.25%; 2.5%; 5% y 10% in the extender. OSS_1.25_; OSS_2.5_; OSS_5_; and OSS_10_=Oil of sesame seeds at 1.25%; 2.5%; 5% y 10% in the extender. TEY=Tris egg yolk base extender. VAP=Average path velocity, VSL=Straight-line velocity, VCL=Curvilinear velocity, STR=Straightness, LIN=Linearity

**Figure-1:**
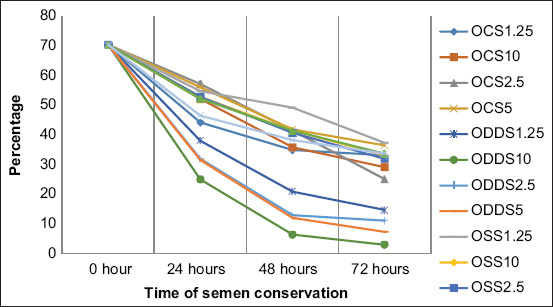
Sperm total motility during semen conservation at 4°C in Merino rams. OCS_1.25_; OCS_2.5_; OCS_5_; and OCS_10_: Oil of cotton seeds at 1.25%; 2.5%; 5%; and 10% in the extender. ODDS_1.25_; ODDS_2.5_; ODDS_5_; and ODDS_10_: Oil of desert date seeds at 1.25%; 2.5%; 5% y 10% in the extender. OSS_1.25_; OSS_2.5_; OSS_5_; and OSS_10_: Oil of sesame seeds at 1.25%; 2.5%; 5% y 10% in the extender. TEY: Tris egg yolk base extender.

**Figure-2:**
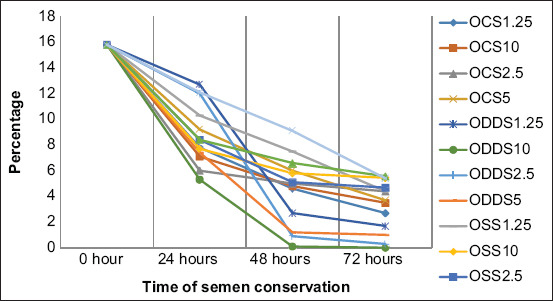
Sperm progressive motility during semen conservation at 4°C in Merino rams. OCS_1.25_; OCS_2.5_; OCS_5_; and OCS_10_: Oil of cotton seeds at 1.25%; 2.5%; 5%; and 10% in the extender. ODDS_1.25_; ODDS_2.5_; ODDS_5_; and ODDS_10_: Oil of desert date seeds at 1.25%; 2.5%; 5% y 10% in the extender. OSS_1.25_; OSS_2.5_; OSS_5_; and OSS_10_: Oil of sesame seeds at 1.25%; 2.5%; 5% y 10% in the extender. TEY: Tris egg yolk base extender.

**Figure-3:**
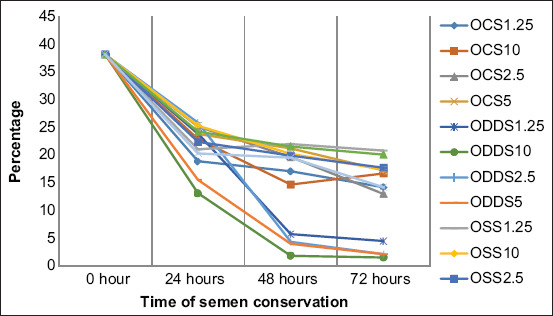
Percentage of spermatozoa with fast movements during semen conservation at 4°C in Merino rams. OCS_1.25_; OCS_2.5_; OCS_5_; and OCS_10_: Oil of cotton seeds at 1.25%; 2.5%; 5%; and 10% in the extender. ODDS_1.25_; ODDS_2.5_; ODDS_5_; and ODDS_10_: Oil of desert date seeds at 1.25%; 2.5%; 5% y 10% in the extender. OSS_1.25_; OSS_2.5_; OSS_5_; and OSS_10_: Oil of sesame seeds at 1.25%; 2.5%; 5% y 10% in the extender. TEY: Tris egg yolk base extender.

**Figure-4:**
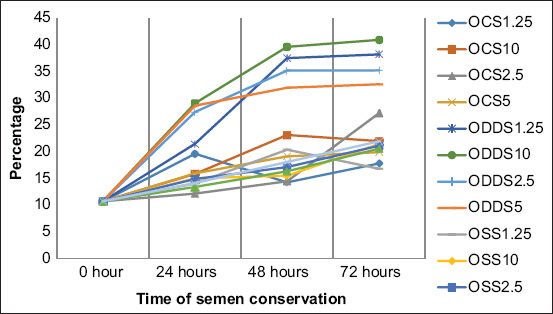
Percentage of static spermatozoa during semen conservation at 4ºC in Merino rams. OCS_1.25_; OCS_2.5_; OCS_5_; and OCS_10_: Oil of cotton seeds at 1.25%; 2.5%; 5%; and 10% in the extender. ODDS_1.25_; ODDS_2.5_; ODDS_5_; and ODDS_10_: Oil of desert date seeds at 1.25%; 2.5%; 5% y 10% in the extender. OSS_1.25_; OSS_2.5_; OSS_5_; and OSS_10_: Oil of sesame seeds at 1.25%; 2.5%; 5% y 10% in the extender. TEY: Tris egg yolk base extender.

When sperm quality was analyzed during the 3 days of preservation, it was observed as an important progressive decrease in the proportion of motile spermatozoa with fast movement and a significant increase of static spermatozoa in all the extenders. Moreover, the formation of two groups of extenders was observed, according to the sperm alteration level, especially near the last day of conservation, where all extenders in the desert date oil base were the most affected negatively compared with the other extenders that preserved sperm quality better (Figures-1-4).

### Second experiment sperm freezing

It is observed in Table-4, sperm frozen in the presence of sesame oil exhibited better motility parameters (mean total motility=44.1±14.0% and mean spermatozoa with fast movements=20.3±9.6%), with the least proportion of spermatozoa moving slowly (47.4±7.5%). In contrast, sperm frozen in BIOXcell and the cottonseed oil-based extender had worse preserved semen quality, although the difference was not statistically significant between these two extenders. The parameters progressive motility, and moderate and static motility yielded better results in the extender containing sesame oil, although this difference was also not significant (p=0.247, 0.09, and 0.128, respectively) from the other diluents. Furthermore, amid the parameters of sperm velocity, only VCL showed a significant difference among all the extenders used to freeze semen, with the best results obtained in sesame oil (Table-5).

**Table-4:** Means values of motility parameters of sperm frozen and thawed in different diluents in Merino rams.

Extenders	MT (%)	MP (%)	Fast (%)	Moderate (%)	Slow (%)	Static (%)
BIOXcell	25.9^b^±11.5	3.1±1.5	9.4±5.5^b^	11.5±6.2	55±3^b^	24.1±10.2
OCS_5_	24.4^b^±12.1	3.2±2.5	8.8±6.4^b^	10.7±5.9	51.6±2.8^ab^	28.9±14.3
OSS_5_	44.1^a^±14	6.3±4.8	20.3±9.6^a^	18.9±5.8	47.4±7.5^a^	13.4±8.7
p-value		0.159		0.059		0.061

Different letters in the same column means statistical difference (p<0.05) between arithmetic means. OCS_5_ and OSS_5_=Oil of cotton seeds and oil of sesame seeds at 5%, respectively. TM=Total motility, PM=Progressive motility

**Table-5:** Means values of velocity parameters of sperm frozen and thawed in different diluents in Merino rams.

Extenders	VAP (mm/seg)	VSL (mm/seg)	VCL (mm/seg)	LIN (%)
BIOXcell	18.8±5.8	8.4±2.6	53.2^ab^±7.4	16±3.5
OCS_5_	17.1±7.1	8.3±3.3	48^b^±11	19.6±2.3
OSS_5_	27.4±9.8	12.4±4.7	68.2^a^±17.6	16.5±1.8
p-value	0.070	0.118	0.086

Different letters in the same column means statistical difference (p<0.05) between arithmetic means. OCS_5_ and OSS_5_=Oil of cotton seeds and oil of sesame seeds at 5%, respectively. VAP=Average path velocity, VSL=Straight-line velocity, VCL=Curvilinear velocity, STR=Straightness, LIN=Linearity

Viability and acrosome integrity tests were conducted by cytometry flux, and there was no significant difference between the three extenders, with p-values of 0.296, 0.334, 0.152, and 0.227, respectively, for live spermatozoa with intact acrosomes, live spermatozoa with disrupted acrosomes, dead spermatozoa with intact acrosomes, and dead spermatozoa with disrupted acrosomes. Nonetheless, the extender supplemented with sesame oil seemed to yield more live spermatozoa with intact acrosome. Moreover, there were apparently fewer dead spermatozoa with disintegrated acrosome and more live spermatozoa with disrupted acrosome in the BIOXcell extender (Table-6).

**Table-6:** Mean values of viability and acrosome integrity of frozen-thawed sperm in Merino breed.

Extenders	LL (%)	LR (%)	UL (%)	UR (%)
BIOXcell	4.1±2.6	56.5±19.8	0.2±0.1	37.4±18.4
OCS_5_	2.8±2.8	42.3±12.8	0.2±0.1	53.3±10.7
OSS_5_	6.1±3.8	44.2±13.5	0.4±0.3	46.3±10.6
p-value	0.146	0.199	0.093	0.107

OCS_5_ and OSS_5_=Oil of cotton seeds and oil of sesame seeds at 5%, respectively. LL=Alive spermatozoa with intact acrosome; LR=Alive spermatozoa with disrupted acrosome; UL=Dead spermatozoa with intact acrosome; UR=Dead spermatozoa with disrupted acrosome

## Discussion

In seminal technology, antioxidants are used commonly to neutralize free radicals in semen, mainly reactive oxygen species, which, at high concentrations, can damage cellular function by producing excessive membrane phospholipid peroxidation, with harmful effects on nuclear and mitochondrial DNA [32]. According to their origin, there are two types of antioxidants: Those produced synthetically, which have been used since 1995, and those formed naturally, antioxidants contained in vegetable oils, used recently to preserve better sperm quality. Many scientific reports have analyzed the effects of vegetable oils on semen quality, either as an animal diet supplementation [33-40] or as a cryoprotectant substance in sperm extenders [20-25,41-44].

Our results demonstrated that sesame seed oil and cottonseed oil added in the Tris-based extender, at concentrations of 1.25-10% and 2.5-5%, respectively, seemed to improve semen quality at 5°C, although that difference was not significant with sperm stored in the extender control. These results are different from those described by others after adding other vegetable oils in the ram extender. Thus, argan oil added at 1% and 5%, respectively, to Tris- and milk-based extenders improved sperm viability, progressive motility, and membrane integrity; at the same time, argan oil decreased the level of spontaneous and induced malondialdehyde and the sperm DNA fragmentation of ram semen preserved at 5°C and 15°C during 48 h, when compared with the control extenders [20].

In the specific case of desert date seed oil, all extenders based on this oil presented the worst seminal quality for all sperm parameters analyzed. That decrease of sperm quality was more significant with the increase in desert date seed oil concentration in the extender; this may be explained by the physical characteristic of desert date seed oil with the lowest density: 0.88 g/mL [45] versus 0.916-0.918 g/mL for cottonseed oil and 0.914-0.919 g/mL for sesame seed oil [46]. This characteristic makes it more difficult to mix desert date seed oil in the extender, as observed during the reconstitution of all these oil-based extenders.

Regarding semen conservation at 4°C, extenders supplemented with sesame oil and 2.5-5% cottonseed oil were not statistically different from the control extender containing egg yolk. This points to the conclusion that vegetable oil fatty acids can replace egg yolk lecithin as an antioxidant to protect spermatozoa against freezing stress.

On the other hand, sperm frozen and thawed in a Tris-based extender supplemented with sesame seed oil preserved sperm motility better than with cottonseed oil supplementation and the BIOXcell extender. This difference could be explained by the higher unsaturated (oleic and linoleic acids) and lower saturated (palmitic and stearic acids) fatty acids in sesame oil than, at least, in cottonseed oil. Indeed, the literature reports that sesame oil contains 71.7-90.5% unsaturated acids and 13.7-17.2% saturated acids [28,47-49] and cottonseed oil contains 58.2-76.8% unsaturated acids and 22.0-40.9% saturated acids in Nergiz *et al*. [50], Kouser *et al*. [51], Shah *et al*. [52]. Moreover, some constitutive components of essential vegetable oils, primarily unsaturated fatty acids, have been proven to have important antioxidant effects [53].

Besides, as BIOXcell is a commercial extender that contains vegetable lecithin instead of egg yolk, it may also have low oleic and linoleic acid levels. This could justify its worst semen motility results compared to sperm frozen and thawed in the sesame oil-based extender, but the difference was not significant for cytometry flow parameters between both extenders.

In general, motility, velocity, viability, and acrosome integrity values obtained in the present study were much lower than those found in the bibliographic review on ram semen conservation; this could be interpreted as a consequence of the initial sperm quality of the ejaculates collected, containing already high levels of immature spermatozoa (32-40%), because of the short rest period of our rams (±3 days) before semen collection and because immature spermatozoa are highly sensitive to cell stress produced during sperm-freezing and thawing processes.

## Conclusion

According to the results obtained, the substitution of egg yolk by 2.5-10% cottonseed oil or 1.25-10% sesame seed oil maintains as much sperm quality as the Tris–egg yolk extender during semen refrigeration at 4°C, but extenders supplemented with oil of the desert date preserved semen poorly at that temperature. In addition, during deep sperm freezing, extender supplementation with 5% of sesame oil resulted in better sperm quality preservation than with cottonseed oil supplementation or the BIOXcell extender.

About 2.5-10% of cottonseed oil and 1.25-10% of sesame seed oil can be used to replace egg yolk contained in a Tris–egg yolk–based extender. Moreover, a Tris–5% sesame seed oil-based extender could be an alternative for freezing ram semen, although these results need to be confirmed with semen collected from rams with appropriate sexual rest.

## Authors’ Contributions

AA: Conceptualization, methodology, investigation, software, data curation, writing original draft, visualization. AE: Methodology, investigation, writing-review and editing, supervision. OP and NAE: Investigation and resources. MG: Writing-review and editing. All authors read and approved the final manuscript.
